# Sexualized drug use, anxiety, depression, and suicidal ideation among men who have sex with men in Pakistan: Results of a multi-center cross-sectional survey

**DOI:** 10.1371/journal.pgph.0006370

**Published:** 2026-05-14

**Authors:** Usman Ali, Syed Azhar Ali, Syed Raza Haider Tirmizi, Tanzil ur Rehman, Tehseen Azar, Babar Yasin, Aftab Tawab Siddiqi

**Affiliations:** 1 Research Consultant, Dostana, Lahore, Pakistanrogram Manager, DMHS, Lahore, Pakistan; 2 Associate Professor of Psychiatry, Shaikh Khalifa Bin Zayed Al Nahyan Hospital, Rawalakot, Pakistan; 3 Program Manager, Dostana, Lahore, Pakistan; 4 CEO Grace Hospital, Rawalpindi, Pakistan; 5 Monitoring and Evaluation Manager, Khawaja Sira Society, Lahore, Pakistan; 6 Associate Professor of Histopathology Combined Military Hospital, Lahore, Pakistan; 7 Postgraduate Resident Psychiatry, King Edward Medical University, Lahore, Pakistan; Institute of Public Health Bengaluru, INDIA

## Abstract

Section 377 of the Pakistan Penal Code penalizes same-sex behavior with life imprisonment. There is a high degree of religious stigma against men who have sex with men (MSM) in Pakistan. The social and legal framework adds to the minority stress for MSM in Pakistan. We conducted a cross-sectional study aimed at examining the association of amphetamine-type stimulant (ATS) use with anxiety and depressive symptoms, and their association with suicidal ideation. Generalized Anxiety Disorder-7 (GAD-7), Patient Health Questionnaire-9 (PHQ-9), and Ask for Suicide Questionnaire-5 (ASQ-5) were used in this study. MSM were recruited from community organizations using convenience sampling from 5 July 2025–5 August 2025. A total of 735 participants identifying as MSM were included in the study. About 10.5% reported ATS use, and 25.4% reported SDU. The prevalence of moderate and severe anxiety symptoms was 12.45% and 14.4%, respectively. The prevalence of moderately severe and severe depressive symptoms was 9.4% and 8.4%, respectively. Around 26.8% of MSM included in the study were non-acute positive, and 6.8% were acute positive for the suicide screen. Having a homosexual partner and ATS use were associated with higher GAD-7 score, β = 1.044 (BCa 95% CI 0.083 to 1.919, p < 0.05) and β = 5.523 (BCa 95% CI 3.932to 7.257, p < 0.001), respectively. ATS use was also associated with an increased PHQ-9 score, β = 6.258 (BCa 95% CI 4.418 to 8.207, p < 0.001), ATS use, PHQ-9, and GAD-7 increase suicidal ideation with the odds ratios of 3.76 (95% CI 1.788 to 7.721), 1.166 (95% CI 1.106 to 1.229), and 1.152 (95% CI 1.093 to 1.214), respectively. Overall, ATS use was high among MSM and associated with the symptoms of anxiety, depression, and suicidal ideation. Comprehensive mental health screening and harm reduction programs for ATS use should be provided to MSM in Pakistan.

## Introduction

Pakistan is one of the twelve countries across the globe where same sex relationships are penalized, including a possible capital punishment [1]. The government of Pakistan has banned gay dating and socializing apps in the country [2]. Alongside a legally invalidating environment, the cultural or religious attitudes are also not conducive to men who have sex with men (MSM) [3–5]. Even though the estimated number of MSM in Pakistan is around 1 million, there is virtually no visibility or human rights enfranchised to the MSM community in the country [6]. As a result, the MSM face various degrees of stigma and discrimination in society. In a large epidemiological study on HIV prevalence, it was found that 31.3% of MSM reported discrimination, and 51.9% reported being physically hurt [7]. In another community survey on HIV stigma, 10% of MSM who were HIV positive reported that they were excluded from family activities due to their sexual orientation [8]. As a result of the stigma and discrimination, MSM face a high degree of stress. In addition, due to the prevalent religious and cultural negative values inherited from society, they may suffer from internalized homophobia [9]. There is a growing HIV epidemic among MSM in the country. Around 5.4% of MSM are living with HIV in Pakistan, which can add to additional stress [7]. The minority stress theory suggests that higher stress due to minority status translates into a higher degree of mental health problems [10].

There are high-risk sexual practices prevalent in the MSM community. Sexualized drug use (SDU) is defined as the use of drugs to facilitate sexual activity. SDU is prevalent in many communities, but especially among MSM, transgender people, and sex workers [11–14]. The popular substances used for SDU include amphetamines [15]. Community organizations working on HIV prevention have reported increasing use of methamphetamines in the local MSM community [16]. These practices carry their risks not only related to increased sexually transmitted infections but also depression, anxiety, and psychosis secondary to substance use [17,18]. Compounding the risk for mental health conditions are the healthcare barriers that the MSM community faces. Around 5.5% of MSM living with HIV feared seeking medical help due to perceived stigma [8]. In another survey, around 6.3% of MSM stated being denied healthcare [7].

Despite the elevated risk of mental health conditions among men who have sex with men (MSM), the mental health status and prevalence of drug use among MSM in Pakistan remain poorly studied. A previous survey assessed the mental health of MSM; however, it included only MSM workers employed in HIV prevention clinics, limiting the generalizability of the findings [19]. To the best of our knowledge, no multicentric prevalence survey has examined drug use and symptoms of common mental health conditions among MSM in the country. The purpose of this study is to identify the prevalence of SDU, ATS use, and mental health outcomes among Pakistani MSM and to investigate the associations between ATS use, anxiety, depression, and suicidal ideation.

## Materials and methods

Ethics Statement: Ethical approval was obtained from Sheikh Khalifa Bin Al Nahyan Hospital (No. 7625SKBZ/ CMH Dated: 4 July 2025).

### Study design, settings, and participant recruitment

A cross-sectional descriptive study was conducted from 5 July 2025–5 August 2025. The study sites included MSM community organizations in Lahore, Sheikhupura, Mandi Bahauddin, Rawalpindi, Karachi, Sialkot, Sargodha, and Rahim Yar Khan. These cities have large MSM communities as identified in the Integrated Biological and Behavioral Surveillance (IBBS) 2016–17 [7]. These organizations are geographically located at sites within these cities where most of the MSM community lives, including MSM brothels and sex work areas. These organizations are led and managed by the MSM community itself and have been providing services for HIV for over a decade with the support of the Global Fund. The workers employed in the field staff identify as MSM. Thus, the participants have been identified by these workers based on personal contacts and routine outreach. In addition, MSM community members also visit the drop-in center of community organizations after referral from other community members. Participants were recruited using convenience sampling. The organization has a confidentiality and privacy policy, which was adhered to during data collection, including no personal identifiable information included in written form, and allocation of unique identity codes for data recording. All data collectors were trained on data collection by the Site Manager. No remuneration was made for data collectors or participants. Written informed consent was taken. Paper-based data was collected. All paper-based data is stored in a lock and key as per organizational policy and discarded after five years as per policy. The safety, privacy, and confidentiality policy of the organization adheres to the Global Fund standards of working in settings that criminalize MSM.

### Inclusion and exclusion criteria

Individuals over 18 years of age, identifying as MSM, gay, or bisexual men, were included. Employees working at community organizations were excluded.

### Study procedures

The study questionnaire consisted of a sociodemographic form, which included questions on drug use, the Generalized Anxiety Disorder-7 (GAD) screening questionnaire, Patient Health Questionnaire (PHQ)-9, and Ask for Suicide Screening Questionnaire (ASQ). Participants were asked ‘Have they used any illicit substance in the last six months (drug use), have they injected an illicit substance at least once in the last six months (injectable drug use), have they use amphetamines (ice) in any form at least once in the last six months (ATS use), and have used any illicit drug during sexual activity at least once in the last six months (SDU). Urdu versions of GAD-7, PHQ-9, and ASQ were used. These tools were previously validated in the Urdu language [20–22]. These tools were also validated for use among HIV communities, including MSM in the National HIV Stigma Index 2.0 and the PLHIV national mental health screening program [8]. PHQ-9 has also been used for MSM living with HIV and MSM community workers [19,23]. GAD-7 and PHQ-9 are rated on a 4-point Likert scale. GAD-7 consists of seven anxiety-specific questions, while PHQ-9 consists of nine depression specific questions. Composite scores are calculated by adding scores on each question. The scores for GAD-7 can vary from 0 to 21, and PHQ-9 scores can range from 0 to 27 [24]. Higher scores on GAD-7 and PHQ-9 are related to higher severity of anxiety and depressive symptoms, respectively. A score of 0-4, 5-9, 10-14, and 15-21 on GAD-7 related to minimal, mild, moderate, and severe anxiety symptoms, respectively. Similarly, on PHQ-9, a score of 0-4, 5-9, 10-14, 15-19, and 20-27 correlates with no or minimal, mild, moderate, moderately severe, and severe depressive symptoms [25]. The Cronbach’s alpha for GAD-7 in our study sample was 0.915, and for PHQ-9 was 0.835. ASQ is a five-question screening tool for suicidal ideation. The Urdu version of the ASQ has been validated in the Pakistani population. The Urdu version of ASQ has a sensitivity of 94.2% and a specificity of 73.9%. The negative predictive value of ASQ is 97.7%, and the positive predictive value is 52.0% [22]. Participants who respond “Yes” to any of the first four ASQ items are considered to screen positive for suicide risk. Those who also answer ‘Yes’ to the fifth question are classified as acute positive, whereas those who answer ‘No’ to the fifth question are classified as non-acute positive for the suicide screen [22].

### Data analysis

Data was entered in Statistical Package for Social Sciences (SPSS), Version 26.0. Descriptive statistics, frequency, percentages, mean, and standard deviation were calculated based on variable type (categorical vs continuous). Prevalence estimates of SDU, injectable drug use, and ATS use, and categories of GAD-7 and PHQ-9 were computed. Multiple linear regression was applied to identify factors associated with symptoms of anxiety and depression on the GAD-7 and PHQ-9 scores, whereas Logistic regression was applied to identify factors associated with symptoms of suicidal ideation. Assumptions of regression, such as Durbin Watson Test, Variance Inflated Ratio (VIF), Tolerance, and normality of residuals, were checked for each regression model [26]. Multicollinearity diagnostics were satisfactory, with tolerance values >0.1 and VIF < 10 for all predictors. Durbin–Watson statistics indicated no autocorrelation for the suicidal ideation model (DW = 1.58), while the anxiety (DW = 1.23) and depressive symptom models (DW = 1.15) suggested possible positive autocorrelation. Because autocorrelation can affect the accuracy of standard errors, we re-estimated the anxiety and depression models using nonparametric bootstrapping (2,000 samples, bias-corrected and accelerated confidence intervals), which provides robust inference in the presence of autocorrelation or heteroskedasticity. We followed the guidelines for Strengthening the Reporting of Observational Studies in Epidemiology (STROBE) [27]. (S1 Checklist). Data is available as supplementary file. (S1 Data).

## Results

A total of 735 participants identifying as MSM were included in the study. The mean age of the study participants was 28.14 years. About 253 (34.4%) of MSM were married to a woman, and 420 (57.1%) were in a homosexual relationship. A total of 208 (28.3%) reported illicit drug use, 25 (3.4%) reported injectable drug use, 77 (10.5%) reported ATS use, and 187 (25.4%) reported SDU. The prevalence of moderate and severe anxiety symptoms was 91 (12.45%) and 106 (14.4%), respectively. Whereas the prevalence of moderately severe and severe depressive symptoms was 69 (9.4%) and 62 (8.4%), respectively. A total of 92 MSM (26.8%) were non-acute suicide screen positive, and 50 (6.8%) were acute positive for the suicide screen. Key sociodemographic and mental health variables are given in Table 1.

**Table 1:** Sociodemographic and mental health variables of MSM in Pakistan, 2025 (n = 735).

Variable	Frequency (Percentage)
Age, mean (S.D)	28.14 (6.8)
Income in PKR, mean (S.D)	40969.17 (51979.37)
**Educational status**	
No formal education	78 (10.6%)
Up to grade 5	143 (19.5%)
Up to grade 10	216 (29.4%)
Up to grade 12	186 (25.3%)
Above grade 12	112 (15.2%)
**Heterosexual marital status**	
Married	253 (34.4%)
Unmarried	482 (65.6%)
**Sexually partnered with a man**	
Yes	420 (57.1%)
No	313 (42.6%)
**Drug use**	
Illicit drug use	208 (28.3%)
Injectable drug use	25 (3.4%)
ATS use	77 (10.5%)
Sexualized drug use in the last six months	187 (25.4%)
GAD-7, mean (S.D)	6.51 (6.20)
**GAD-7 categories**	
No anxiety symptoms	357 (48.6%)
Mild anxiety symptoms	181 (24.6%)
Moderate anxiety symptoms	91 (12.45%)
Severe anxiety symptoms	106 (14.4%)
PHQ-9, mean (S.D)	8.13 (6.65)
**PHQ-9 categories**	
No depressive symptoms	248 (33.7%)
Mild depressive symptoms	249 (33.9%)
Moderate depressive symptoms	107 (14.6%)
Moderately severe depressive symptoms	69 (9.4%)
Severe depressive symptoms	62 (8.4%)
**ASQ categories**	
Negative suicide screening	488 (66.4%)
Non-acute positive screening	197 (26.8%)
Acute positive screening	50 (6.8%)

Multiple linear regression was applied to identify factors associated with symptoms of anxiety on GAD-7 scores. The final model had an adjusted R-squared of.1034, p < 0.001. Increased education was associated with increased score on GAD 7, β = 705 (BCa 95% Confidence Interval, CI 0.309 to 1.152, p < 0.001). Similarly, having a homosexual partner and ATS use were associated with higher GAD-7 score, β = 1.044 (BCa 95% CI 0.083 to 1.919, p < 0.05) and β = 5.523 (BCa 95% CI 3932to 7.257, p < 0.001), respectively. Having a marital relationship with a woman and an increased salary were associated with reduced GAD-7 score, β = - 1.261 (BCa 95% CI -2.304 to -0.124, p < 0.05) and β = -0.086 (BCa 95% CI -0.177 to -0.016, p < 0.05), respectively. Results of multiple linear regression for GAD-7 score are given in Table 2.

**Table 2:** Factors associated with anxiety symptoms among MSM (n = 735).

Variable	B	Standard error	P-value	BCa 95% Confidence Interval	
				Lower limit	Upper limit
Intercept	2.877	1.060	.008	.717	5.081
Education	.705	.206	.001**	.309	1.152
Having a marital relationship with a woman	-1.261	.564	.027*	-2.304	-.124
Sexually partnered with a man	1.044	.453	.021*	.083	1.919
ATS use	5.523	.783	.000***	3.932	7.257
Age (in years)	.063	.037	.090	-.010	.131
Income (10,000 PKR unit)	-.086	.046	.043*	-.177	-.016

*Significant at p < .05; ***Significant at p < .001

Multiple linear regression was used to identify the factors associated with symptoms of depression on the PHQ-9 score. The model explained a modest amount of variance (adjusted R-squared 0.107, p < 0.001). Increased education and ATS use were associated with increased PHQ-9 score, β = 0.637 (BCa 95% CI 0.210 to 1.064, p < 0.01) and β = 6.258 (BCa 95% CI 4.418 to 8.207, p < 0.001), respectively. Whereas having a marital relationship with a woman were associated with reduced PHQ-9 scores, β = -1.722 (BCa 95% CI -2.891 to -0.528, p < 0.01) and β = -0.100 (BCa 95% CI -0.212 to -0.023, p < 0.05), respectively. Results are given in Table 3.

**Table 3:** Factors associated with depressive symptoms among MSM (n = 735).

Variable	B	Standard error	P-value	BCa 95% Confidence Interval	
				Lower limit	Upper limit
Intercept	4.833	1.204	.000***	2.406	7.468
Education	.637	.218	.004**	.210	1.064
Having a marital relationship with a woman	-1.722	.574	.004**	-2.891	-.528
Sexually partnered with a man	.769	.476	.105	-.132	1.636
ATS use	6.258	.951	.000***	4.418	8.207
Age (in years)	.068	.041	.089	-.011	.145
Income (10,000 PKR unit)	-.100	.048	.032*	-.212	-.023

*Significant at p < .05; **Significant at p < .01; ***Significant at p < .001

We used binary logistic regression to identify the factors associated with symptoms of suicidal ideation on ASQ-5. The model had a Cox & Snell R-squared of 0.398, p < 0.001. The Hosmer–Lemeshow test indicated good fit (χ² = 14.56, p > 0.05). The odds ratio of suicidal ideation was significant for marital relationship with a woman, Adjusted Odds Ratio (AOR)=0.435, 95% CI 0.263 to 0.788, p < .01. ATS use, PHQ-9, and GAD-7 were associated with the odds ratio of 3.76 (95% CI 1.788 to 7.721, p < 0.001), 1.166 (95% CI 1.106 to 1.229, p < 0.001), 1.152 (95% CI 1.093 to 1.214, p < 0.001), respectively. Results of binary logistic regression are given in Table 4.

**Table 4:** Factors associated with suicidal ideation among MSM (n = 735).

Variable	Odds ratio	Standard error	z	P value	95% confidence interval	
					Lower limit	Upper limit
Intercept	-4.167	0.015	39.335	.000		
Education (Reference, no formal education)			5.534	.237		
Up to grade 5	2.117	.442	2.876	.090	.890	5.039
Up to grade 10	1.415	.421	.680	.410	.620	3.230
Up to grade 12	1.352	.431	.490	.484	.581	3.146
Above grade 12	2.315	.480	3.057	.080	.903	5.933
Having a marital relationship with a woman	.455	.280	7.892	.005**	.263	.788
Sexually partnered with a man	1.546	.234	3.464	.063	.977	2.445
ATS use	3.716	.373	12.376	.000***	1.788	7.721
PHQ-9 score	1.166	.027	32.791	.000***	1.106	1.229
GAD-7 score	1.152	.027	27.957	.000***	1.093	1.214
Age (in years)	1.016	.019	.700	.403	.979	1.053
Income (10,000 PKR unit)	1.023	.020	1.358	.244	.985	1.063

Significant at p < .05; **Significant at p < .01; ***Significant at p < .001

## Discussion

Our study is the first quantitative study on mental health and drug use aspects of MSM in the country. Previously, stigma and discrimination have been explored in two large surveys on HIV, with one exclusively recruiting people living with HIV, with a sub-sample of MSM [7,8]. In our study, the prevalence of illicit drug use was 28.3%. The prevalence of illicit drug use among MSM has not been reported in the IBBS Report 2017. In our study, 25.4% of MSM had reported SDU in the last six months, whereas in IBBS, 47.7% of MSM reported SDU in the last twelve months [7]. The difference in these estimates can be explained by the different timeframes used in the two surveys. Further, the IBBS recruited MSM from hotspots or areas of sex work as well as snowballing, whereas in our study, MSM were recruited from HIV prevention organizations. Thus, the included sample in our study may be more aware of the consequences of drug use and may differ systematically from the sample in IBBS. Another plausible explanation can be reporting bias, as MSM may not disclose their drug use patterns to the data collectors, who were also their social workers. In a meta-analysis on SDU among MSM in South and East Asia, the pooled prevalence of SDU was 13% [14]. Similarly, in our study, the prevalence of injectable drug use was 3.4%, whereas the 12-month prevalence of injectable drug use was 4.4% in the previous survey [7]. The surge of ATS use, especially in the context of MSM, is a recent phenomenon. ATS use was not recorded before in the Drug Survey of Pakistan 2013 [28]. This nationally representative survey reported 19,000 people who use amphetamines across the country. The estimates include prescription and non-prescription amphetamines. The prevalence of ATS use among MSM has not been reported in the Drug Survey of Pakistan. A recent report from a non-profit organization reported that the prevalence of ATS was 80.2% among MSM and transgender people. However, the study had methodological limitations, including purposively approaching people who use drugs and a small sample size [16]. In another survey on the acceptability of HIV pre-exposure prophylaxis, 19.7% of the MSM reported using ATS during sex in the last six months, whereas in our study, 10.5% reported using ATS in any setting [29]. In the IBBS survey from Bangladesh, the prevalence of ATS use in the last six months among MSM was 1.7% [30]. In a recently conducted qualitative study, study participants highlighted poor mental health, such as body image issues, stress, anxiety, and depression, as key drivers for ATS use among MSM and as female sex workers (FSWs) and transgender people [31].

In terms of symptoms of anxiety among MSM, our study showed that 26.85% had anxiety of at least moderate severity. Pitonak et al., have conducted an epidemiological survey on the prevalence of mental health conditions among sexual and gender minorities in the Czech Republic. The employed Mini International Neuropsychiatric Interview (MINI) [32]. According to their study, 15.91% of sexual and gender minorities had an anxiety disorder. In the same study, the mean GAD-7 score for homosexual men and women was 3.5, among bisexual men and women was 4.63, and among sexually diverse men and women was 3.7 [32]. However, in our study, the mean GAD-7 score was 6.51, which is higher than the estimates reported by Pitonak [32]. Field et al., have studied anxiety among MSM living with HIV in the Netherlands [33]. The study has found a 25.1% prevalence of anxiety symptoms, which is lower than our estimates. Plausible reasons for these results could include more validating laws in the Netherlands for MSM. However, it should be noted that in the same study, the prevalence of chemsex was higher, i.e., 48.5%. Resembling findings from our study, age was not associated with an increase in anxiety symptoms [33]. However, in contrast to findings from our study, chemsex (defined as use of mephedrone, methamphetamine, GHB/GBL, cocaine, ketamine, speed, ecstasy/MDMA, and/or other designer drugs) was also not associated with an increase in anxiety symptoms, while, in our study, GAD-7 score had been associated by ATS with a large beta-coefficient.

In terms of depressive symptoms, the prevalence of depressive symptoms that are at least moderately severe was 17.8%. In a previous study, among MSM and transgender health workers in Pakistan, the prevalence of moderately severe and severe depressive symptoms was 9.89% and 7.6%, respectively. In a meta-analysis, the global prevalence of depressive symptoms among MSM was 35% [34]. The analysis showed a higher prevalence of depressive symptoms among MSM from Asian countries and the lowest in Europe [34]. In terms of factors associated with depressive symptoms, higher education was associated with increased depressive symptoms. Whereas ATS use was associated with an increase in depressive symptoms. These findings contradict findings by Field, who report no association between education and depressive symptoms [33]. The odds ratio of a potential depressive disorder for those using chemsex (including ATS use) was 0.40 in the cited study [33].

Consistent with findings from Pitonak, who reported a 25% prevalence of suicidal ideation, in our study, 26.8% were positive for a non-acute positive suicide screen [32]. There are two other reports of suicidal ideation among MSM in Pakistan [19,35]. Both studies were conducted among people employed in the HIV community organization. The prevalence of suicidal ideation in one study was reported to be 8.8%. Whereas the prevalence of deliberate self-harm or non-suicidal self-injury was 5.5% [19]. In a more recently conducted study, 27.1% of the participants stated a history of suicidal ideation, which is close to the estimates in the present study. The study also reported a 13.8% prevalence of self-harm [35]. In a study conducted on MSM in Nepal, the prevalence of suicidal ideation was around 40%, while 21.6% had a suicidal attempt [36]. The study also reported an increased suicidal ideation with higher education (OR 2.9) and suicidal plan (OR 2.2) [36]. In our study, there was no association between suicidal ideation and education status, though a positive association existed for anxiety and depressive symptoms. ATS was associated with increased risk of suicidal ideation, with an odds ratio of 3.76. This finding is consistent with a meta-analysis, which reported an odds ratio of 2.2 for suicidal ideation and 3.6 for suicidal attempt.

### Limitations of the study

The study had various limitations that warrant caution for interpretation. The study was conducted at eight sites across Pakistan. The study sample consisted mostly of MSM in specific cities of Punjab and Karachi. Pakistan has four provinces, and MSM from interior Sindh, Khyber Pakhtunkhwa, and Balochistan were not included in the study. Therefore, the findings are not generalizable to MSM across the entire country The use of convenience sampling may have introduced selection bias; thus, the findings have limited generalizability in cities where the study was conducted. The data collectors were also service providers and social workers of the participants, which can lead to reporting bias, as MSM may not feel comfortable disclosing drug use to their service providers. As the study tools used are for screening only, and given the high prevalence of drug use, it cannot be ascertained whether the symptoms reflect a primary psychiatric disorder or symptoms secondary to drug use. Furthermore, the variance explained by regression models was only modest; thus, there are other explanatory factors associated with symptoms of anxiety and depression, which were not studied in our study. A consistent finding in our study was that MSM who had a heterosexual marriage had significantly lower GAD-7 and PHQ-9 scores and a lower odds ratio for suicidal ideation. We have not computed whether this relationship varies among gay men, bisexual men, or men with same sex behavior.

## Conclusion

The study highlights a high degree of psychiatric morbidity among MSM. SDU and ATS use are prevalent in the MSM community. ATS is also correlated with the symptoms of anxiety and depression, as well as suicidal ideation. While the findings have limited generalizability to MSM across Pakistan and even in the cities where the study was conducted, it highlights report of high prevalence of symptoms of depression, anxiety, and suicidal ideation. There is a need to integrate comprehensive mental health and harm reduction programs. Currently, HIV services are provided by community organizations. Staff at these organizations need to be sensitized on mental health as well as equipped with screening for mental health conditions and providing psychosocial support. There are no referral pathways from these organizations to primary and secondary healthcare for mental health conditions. A clear referral pathway and further sensitization of mental health service providers on gender and sexuality can help in reducing barriers to mental health care [37]. Further research with representation from other provinces, as well as the effectiveness of the community mental health program in providing services, is warranted.

## Supporting information

S1 Checklist STROBE checklist. (DOC)

S1 Data Data file. (XLSX)
